# Embryonic origin of the gnathostome vertebral skeleton

**DOI:** 10.1098/rspb.2017.2121

**Published:** 2017-11-22

**Authors:** Katharine E. Criswell, Michael I. Coates, J. Andrew Gillis

**Affiliations:** 1 Department of Organismal Biology and Anatomy, University of Chicago, Chicago, IL, USA; 2 Department of Zoology, University of Cambridge, Cambridge, UK; 3 Marine Biological Laboratory, Woods Hole, MA, USA

**Keywords:** vertebral skeleton, skate, somite, notochord, vertebrae, evolution

## Abstract

The vertebral column is a key component of the jawed vertebrate (gnathostome) body plan, but the primitive embryonic origin of this skeleton remains unclear. In tetrapods, all vertebral components (neural arches, haemal arches and centra) derive from paraxial mesoderm (somites). However, in teleost fishes, vertebrae have a dual embryonic origin, with arches derived from somites, but centra formed, in part, by secretion of bone matrix from the notochord. Here, we test the embryonic origin of the vertebral skeleton in a cartilaginous fish (the skate, *Leucoraja erinacea*) which serves as an outgroup to tetrapods and teleosts. We demonstrate, by cell lineage tracing, that both arches and centra are somite-derived. We find no evidence of cellular or matrix contribution from the notochord to the skate vertebral skeleton. These findings indicate that the earliest gnathostome vertebral skeleton was exclusively of somitic origin, with a notochord contribution arising secondarily in teleosts.

## 1. Introduction

The presence of vertebrae is a defining feature of the vertebrate body plan. A vertebral skeleton may consist of a series of paired neural arches that cover the spinal cord, paired haemal arches that enclose the caudal artery and vein, and, in many jawed vertebrates (gnathostomes), a series of centra that replace the notochord as the predominant support structure. Vertebral centra are highly variable in terms of morphology and tissue composition, and likely evolved independently in many different gnathostome lineages, including tetrapods, teleost fishes and cartilaginous fishes [1]. This apparent evolutionary convergence raises questions about the embryonic origin of vertebral skeletal elements across gnathostomes.

In tetrapods, all components of the vertebral skeleton derive from somites: transient, bilateral blocks of segmented paraxial mesoderm that form dorsally within the embryonic trunk. Somites are partitioned into dorsal and ventral subdivisions that give rise to trunk connective tissue and musculature (‘dermomyotome’) and skeletal tissues (‘sclerotome’), respectively. Cell lineage tracing experiments using chick–quail chimaeras [2–5] and fluorescein–dextran injections or grafts from GFP-transgenic donor embryos in axolotl [6] have shown a fully somitic origin of the vertebral skeleton in these taxa, with somite-derived cells recovered in developing arches and nascent cartilage of the centra.

Conversely, in teleost ray-finned fishes, the vertebral skeleton appears to have a dual embryonic origin, with contributions from both paraxial mesoderm and the notochord. Teleost vertebral centra consist of an inner layer (the chordacentrum) and an outer layer, both composed of bone that forms by intramembranous ossification [7]. The chordacentrum of teleosts forms first, by secretion of bone matrix proteins (e.g. SPARC, type I collagen) from ‘chordoblast’ cells that reside within the notochord epithelium [8–10]. In zebrafish, *in vitro* assays have shown that cultured notochord cells have the capacity to secrete bone matrix, and ablation experiments have demonstrated that in the absence of notochord, chordacentra fail to form [11]. Teleost chordacentra are subsequently surrounded by a relatively late-developing layer of paraxial mesoderm-derived membrane bone [7,12]. Additionally, zebrafish mutants with somite patterning defects possess normally developing chordacentra, but exhibit profound neural and haemal arch defects, indicating the likely paraxial mesodermal origin of arch tissues [11,13,14].

**Figure 1.**
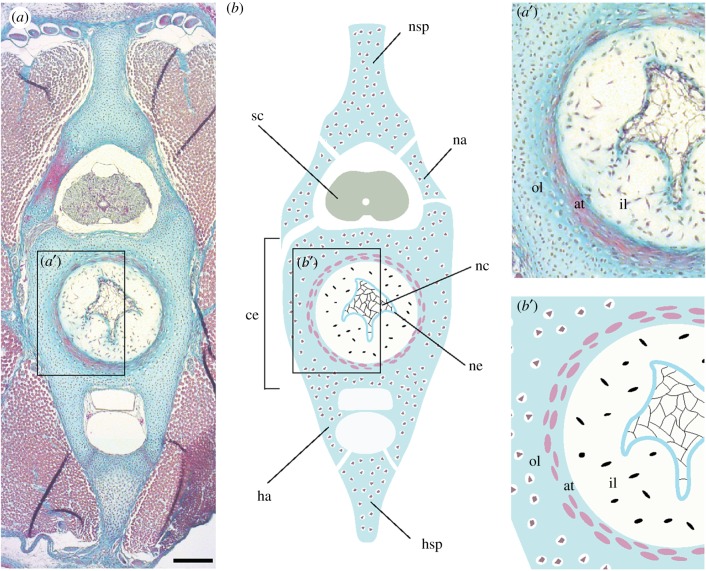
(*a*) Cross section through a skate caudal vertebra (stained with Masson's trichrome); (*a*′), magnified cross section illustrating the three layers of the centrum; (*b*) schematic illustrating the components and tissues of the skate vertebra; (*b*′) schematic of the tri-layered centrum. at, areolar tissue; ce, centrum; ha, haemal arch; hsp, haemal spine; il, inner layer of the centrum; na, neural arch; nc, notochord; ne, notochord epithelium; nsp, neural spine; ol, outer layer of the centrum; sc, spinal cord. Scale bar, 200 µm.

To determine whether the dual origin of vertebral centra is a teleost-specific feature of the vertebral skeleton, or a general feature for gnathostomes that has been lost in tetrapods, data on the embryonic origin of vertebrae from an outgroup to the bony fishes (i.e. Osteichthyes: the group that includes tetrapods and teleosts) are needed. Cartilaginous fishes (Chondrichthyes: sharks, skates, rays and holocephalans) occupy a key phylogenetic position as the sister group to the bony fishes, and data from this lineage may therefore be used to help infer primitive developmental conditions for the last common ancestor of gnathostomes. We have previously shown that vertebrae in the little skate (*Leucoraja erinacea*) each consist of a dorsal neural spine, two sets of dorsal cartilages that enclose the spinal cord (neural and intercalary arches), a single haemal arch and spine extending ventrally, and a tri-layered centrum (figure 1) [15]. Here, we use somite and notochord fate mapping experiments, as well as mRNA *in situ* hybridization for genes encoding skeletal matrix proteins, to test the embryonic origin of the skate vertebral skeleton. We show that all components of the skate vertebral skeleton derive from paraxial mesoderm, with no evidence for cellular or matrix contributions from the notochord. When considered alongside data from bony fishes, our findings point to a general and probably primitive paraxial mesodermal origin of the vertebrate column in jawed vertebrates.

## 2. Material and methods

### (a) Somite fate mapping

**Figure 2.**
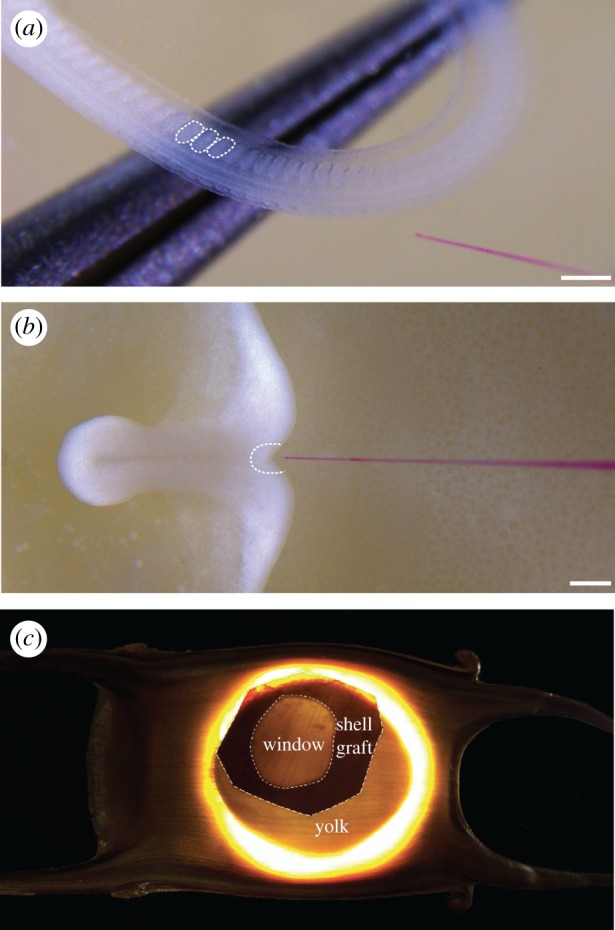
Microinjection of skate embryos with CM-DiI. CM-DiI labelling of (*a*) somites at S24 (three somites are highlighted with dashed lines) and (*b*) notochord progenitor cells at S14 (with the ‘notochord triangle’ of Ballard *et al*. [16] outlined). (*c*) Sealing of a windowed skate egg with donor egg shell. Scale bars, 200 µm.

*Leucoraja erinacea* embryos were obtained from the Marine Biological Laboratory (MBL) in Woods Hole, MA, and kept in a flow-through sea table at approximately 16°C until S24. A flap was cut in the egg case using a razor blade, and the embryo and yolk were transferred to a Petri dish. Embryos were anaesthetized in a solution of MS-222 (100 mg l^−1^ ethyl 3-aminobenzoate methanesulfonate—Sigma-Aldrich) in seawater. CellTracker CM-DiI (Thermofisher) (5 µg µl^−1^ in ethanol) was diluted 1 : 10 in 0.3 M sucrose and injected into the ventral portions of the somites (one to three injections per embryo) using a pulled glass capillary needle and a Picospritzer pressure injector (figure 2*a*). Embryos were then replaced in their egg cases and returned to the sea table to develop for approximately 7 or 12 weeks. Embryos were then fixed with 4% PFA, as described in Criswell *et al*. [15].

### (b) Notochord fate mapping

Embryos were kept as described above until S14, at which point a small window was cut in the egg case over the embryo. CM-DiI was microinjected into the notochord triangle as described above (figure 2*b*). The window was then sealed with donor eggshell and Krazy Glue™ gel (figure 2*c*), and eggs were returned to the sea table to develop for an additional 16–18 weeks prior to fixation (as described in Criswell *et al*. [15]).

### (c) Validation of CM-DiI injection placement

To verify the correct placement of CM-DiI injections, three somite-injected embryos were fixed immediately post-injection, and three notochord-injected embryos were fixed 5 days post-injection (dpi). Embryos were fixed in 4% paraformaldehyde in PBS overnight at 4°C, rinsed 3 × 15 min in PBS and stained with DAPI at 1 µg ml^−1^ overnight at room temperature. Somite-injected embryos were imaged on a Zeiss lightsheet microscope and notochord-injected embryos were imaged on Zeiss lightsheet or LSM 780 confocal microscopes.

### (d) Histology and mRNA *in situ* hybridization

CM-DiI-labelled *L. erinacea* embryos were embedded in paraffin wax and sectioned at 8 µm thickness as described in O'Neill *et al*. [17] for histological analysis. Prior to embedding, embryos were demineralized in 10% EDTA (ethylenediaminetetraacetic acid) for 14 days. Histochemical staining was performed following the Masson's trichrome protocol of Witten and Hall [18]. *In situ* hybridization experiments for *Col1a1* (GenBank accession number MG017616) and *SPARC* (GenBank accession number MG017615) were performed on sections as described in O'Neill *et al*. [17], with modifications according to Gillis *et al*. [19].

## 3. Results

### (a) Somitic contribution to all components of the skate vertebral skeleton

**Figure 3.**
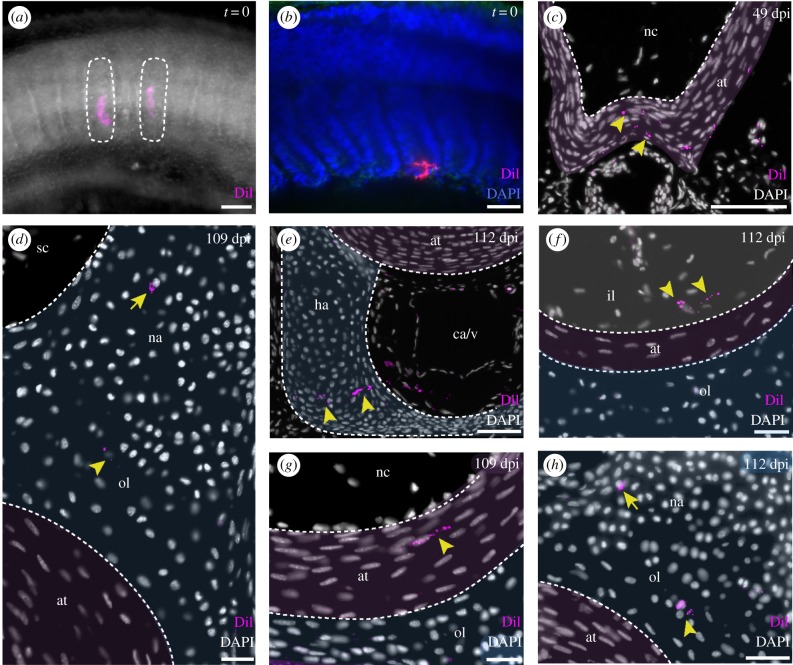
Somitic contribution to the skate vertebral skeleton. (*a*) Two CM-DiI injections in ventral somites; (*b*) confocal image confirming the placement of the dye immediately post-injection in sagittal section; (*c*) CM-DiI-labelled cells (indicated by yellow arrowheads) distributed within the spindle-shaped cells of the areolar tissue (at) at 49 dpi (false coloured pink); (*d*) CM-DiI-labelled chondrocytes in the neural arch (na, indicated by yellow arrow) and outer layer of centrum cartilage (ol, indicated by yellow arrowhead) at 109 dpi (cartilage false coloured blue); (*e*) CM-DiI-labelled cells in the haemal arch at 112 dpi (ha, false coloured blue); (*f*) CM-DiI-labelled chondrocytes (indicated by yellow arrowheads) in the inner layer of the centrum at 112 dpi (il, false coloured white); (*g*) CM-DiI-labelled cells (indicated by yellow arrowhead) in the areolar tissue, the middle layer of the centrum at 109 dpi (at, false coloured pink); (*h*) CM-DiI-labelled chondrocytes in the outer layer of the centrum (ol, indicated by yellow arrowhead) and in the neural arch (indicated by yellow arrow) at 112 dpi (na, false coloured blue). ca/v, caudal artery and vein; nc, notochord; sc, spinal cord. Scale bars, 100 µm.

To test for somitic contribution to the skate vertebral skeleton, we microinjected CM-DiI into ventral portions of the somites (i.e. the presumptive sclerotome—figure 3*a*) of skate embryos at stage (S) 24 (Ballard *et al*. [16]). Focal labelling of the somites (with no notochordal contamination) was confirmed by light sheet microscopy, in embryos fixed immediately post-injection (figure 3*b*; *n* = 3). By 50–52 dpi (S31), spindle-shaped cells of the developing areolar tissue of the centrum surround the notochord, and preskeletal mesenchyme has condensed around the neural tube and caudal artery and vein. In all embryos analysed at this stage (*n* = 5), CM-DiI was recovered in the spindle-shaped cells of the developing areolar tissue (figure 3*c*), indicating their somitic origin.

By 109 dpi (S34), vertebrae are fully developed, with neural, intercalary and haemal arches, and a tri-layered centrum (figure 1). In embryos analysed at this stage (*n* = 4), CM-DiI-positive cells were recovered throughout the vertebral skeleton. CM-DiI-positive cells were recovered in the cartilage of the neural (*n* = 3 vertebrae in three embryos) and haemal arches (*n* = 6 vertebrae in four embryos; figure 3*d*,*e*), as well as in the inner layer of cartilage (figure 3*f*; *n* = 2 vertebrae in two embryos), the middle areolar tissue (figure 3*g*; *n* = 3 vertebrae in three embryos) and the outer cartilage of the centrum (figure 3*h*; *n* = 3 vertebrae in three embryos). Taken together, these findings demonstrate somitic contribution to all major components of the skate vertebral skeleton.

### (b) No evidence for notochordal contribution to the vertebral skeleton in skate

**Figure 4.**
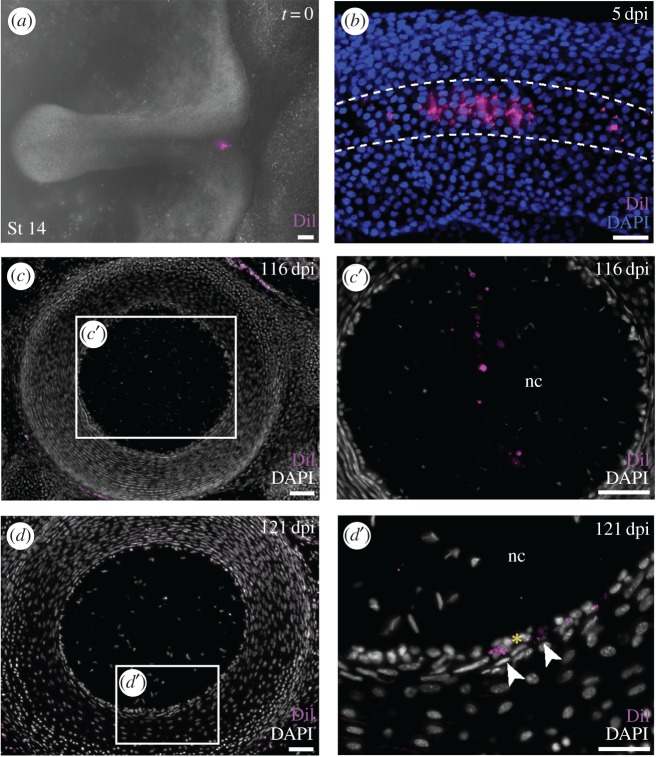
No cellular contribution from the notochord to the skate vertebral skeleton. (*a*) CM-DiI injection of the notochord triangle of a skate embryo at S14; (*b*) confocal image of a skate embryo at 5 dpi, showing CM-DiI-labelled cell in the notochord; (*c*) a section through the notochord at 116 dpi, showing CM-DiI-positive notochord cells at 10×; (*c*′) higher magnification view of the inset box in (*c*); (*d*) CM-DiI-positive cells in the notochord epithelium; (*d*′) higher magnification view of the inset box in (*d*). Yellow asterisk indicates notochord epithelium. Scale bars, 100 µm.

To test for cellular contributions of the notochord to the skate vertebral skeleton, we conducted a series of notochord fate-mapping experiments. In cartilaginous fishes, the notochord derives from a small triangular region of progenitor cells (the ‘notochord triangle’) that appears at the posterior margin of the blastodisc at S12 [16]. We focally labelled the notochord triangle of skate embryos with CM-DiI at S14 (figure 4*a*), and we confirmed localization of the dye to the notochord at 5 dpi (approx. S17) using confocal microscopy. In three embryos examined at S17, CM-DiI was found either only in the notochord (*n* = 2), or in the notochord and neural tissue (*n* = 1) (figure 4*b*). In no cases were CM-DiI-labelled cells detected in the paraxial mesoderm.

We therefore labelled the notochord triangles of several skate embryos at S14, and reared these embryos to 116–129 dpi (S34—at which point the vertebral skeleton has fully differentiated). CM-DiI was recovered within the notochord (figure 4*c*,*c*′) and the notochord epithelium (figure 4*d*,*d*′) of the intervertebral regions of the axial column (*n* = 5). In three embryos, CM-DiI-positive cells were recovered in the remnants of notochord epithelium that persist in the centre of the centrum, where the notochord is almost completely replaced by inner layer centrum cartilage, but no CM-DiI-positive chondrocytes were recovered in the inner layer of cartilage itself. No CM-DiI-labelled chondrocytes were observed in any other components of the axial column. These experiments, therefore, provide no evidence for a cellular contribution from the notochord to the vertebral skeleton.

**Figure 5.**
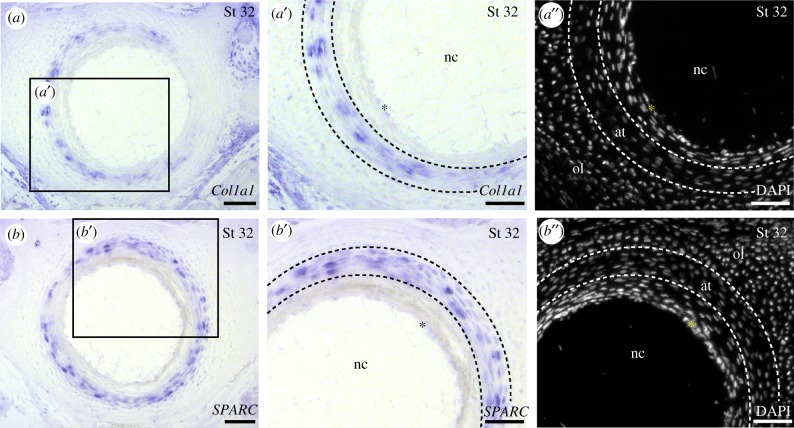
The notochord is not a source of bone-like tissue in skate vertebral centra. (*a*) *Col1a1* is expressed in the areolar tissue of the developing centrum; (*a*′) a higher-magnification image of *Col1a1* expression; (*a*′′) DAPI staining of the same section as depicted in (*a*′), showing the boundary between areolar tissue and the notochord epithelium (yellow asterisk); (*b*) *SPARC* is expressed in the areolar tissue of the developing centrum; (*b*′) a higher-magnification image of *SPARC* expression, and (*b*′′) DAPI staining of the same section as depicted in (*b*′), showing the boundary between areolar tissue and the notochord epithelium (yellow asterisk). at, areolar tissue; nc, notochord; ol, outer layer. Scale bars, 100 µm.

In teleosts, chordoblast cells within the notochord epithelium secrete matrix components that make up the acellular bone of the chordacentrum. Though skates do not possess a chordacentrum, the areolar tissue of the skate centrum does mineralize, and at its origin sits adjacent to the notochord epithelium [15]. To test whether notochord epithelial cells contribute matrix components to centrum tissue in skate, we characterized the expression of genes encoding the bone matrix proteins Col1a1 and SPARC in developing skate centra. We did not detect transcription of *Col1a1* (figure 5*a*) or *SPARC* (figure 5*b*) in the notochord epithelium. Rather, these transcripts localized to the spindle-shaped cells of the areolar tissue (figure 5*a,b*). These findings suggest that the paraxial mesoderm-derived cells of the areolar tissue itself—and not the notochord epithelium—are the source of extracellular matrix of the mineralized tissue of the skate vertebral centrum.

## 4. Discussion

Our somite fate mapping experiments demonstrate that presumptive sclerotome contributes to all components of the vertebrae in skate, including the neural and haemal arches, and all tissues of the tri-layered vertebral centrum. While it is possible that DiI could diffuse through the extracellular matrix after injection to contaminate tissues adjacent to the intended target (e.g. notochord), we have controlled for this possibility by imaging a subset of embryos shortly after injection to validate the precision of our labelling, and by performing complementary notochord fate mapping experiments. In the latter, we find that CM-DiI labelling of notochord progenitor cells resulted exclusively in labelling of the notochord and the notochord epithelium, with no contribution to vertebral tissues. In teleost fishes, chordoblast cells within the notochord epithelium express genes encoding the bone matrix proteins type I collagen and SPARC [10,20–22], and are probably the source of bone matrix for the earliest layer of the vertebral centrum [11,23–28]. As skates also possess a mineralized layer within their vertebral centra, we sought to test for expression of *Col1a1* and *SPARC* during skate vertebral development by mRNA *in situ* hybridization. We found these genes to be expressed exclusively within the somitically derived spindle-shaped cells of the areolar tissue (the precursor to the mineralized middle layer of the centrum—Criswell *et al*. [15]), and not in the notochord epithelium. These findings suggest that the cells and matrix components of the skate vertebral centrum are entirely of paraxial mesodermal origin.

**Figure 6.**
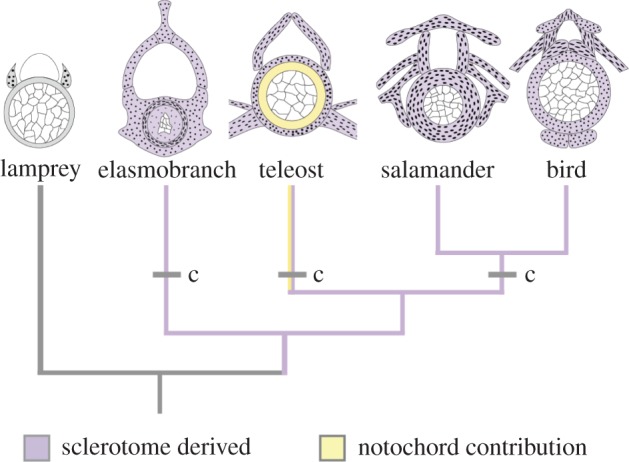
Embryonic origins of the vertebral skeleton across gnathostomes. Representative sections of lamprey, skate, teleost, salamander and bird vertebrae, with paraxial mesodermal derivatives indicated by purple, and notochord derivatives indicated by yellow. Grey bars indicate independent originations of centra. Schematics redrawn after Goodrich [29] (lamprey), Criswell *et al*. [15] (skate) and MacBride [30] (teleost, salamander and bird).

When considered alongside data from bony fishes, our demonstration of a somitic origin of the vertebral skeleton of skates suggests that this tissue was likely the sole, primitive source of vertebral skeletal tissues in gnathostomes, with a notochord contribution to centrum bone representing a derived condition of teleost fishes (figure 6). Evidence from early fossil jawed and jawless fishes strongly suggests that the vertebral skeleton in the last common ancestor of gnathostomes consisted simply of a series of neural arches and a persistent notochord, with no centra [31–34]. Several gnathostome lineages, including elasmobranch cartilaginous fishes, teleosts and tetrapods, subsequently evolved centra independently of one another [1]. At their origins, the vertebral centra of elasmobranchs and tetrapods derived entirely from paraxial mesoderm [3,6,12], but an inner layer of notochord-derived acellular bone was incorporated into the centrum with the independent origin of teleost centra.

It is not yet clear, however, if this specialized condition of teleosts is unique among ray-finned fishes. Despite recent changes to phylogenetic patterns [35], vertebral centra very likely evolved independently in multiple non-teleost ray-finned fish lineages (e.g. in gars and bichirs [1,36,37]). But, it is unclear whether the notochord contributes tissue to the different forms of centra observed in these taxa. Comprehensive analyses of the embryonic origins of vertebral tissues in strategically selected fish taxa are needed to better resolve the evolutionary and developmental assembly of the diverse array of axial skeletons, arguably the key characteristic, of vertebrates in general.

## Data accessibility

The sequence data associated with the genes in this study are available on GenBank (*Col1a1* accession number MG017616 and *SPARC* accession number MG017615).
